# Constitutive activation of nitrate reductase in tobacco alters flowering time and plant biomass

**DOI:** 10.1038/s41598-021-83797-7

**Published:** 2021-02-19

**Authors:** Jianli Lu, Niharika N. Chandrakanth, Ramsey S. Lewis, Karen Andres, Lucien Bovet, Simon Goepfert, Ralph E. Dewey

**Affiliations:** 1 Department of Crop and Soil Sciences, North Carolina State University, Raleigh, NC USA; 2 PMI R&D, Philip Morris Products S.A., Neuchatel, Switzerland; 3 Present Address: ClinChoice Inc., Fort Washington, PA USA

**Keywords:** Biotechnology, Plant sciences

## Abstract

Pyridine alkaloids produced in tobacco can react with nitrosating agents such as nitrite to form tobacco-specific nitrosamines (TSNA), which are among the most notable toxicants present in tobacco smoke. The market type known as burley tobacco is particularly susceptible to TSNA formation because its corresponding cultivars exhibit a nitrogen-use-deficiency phenotype which results in high accumulation of nitrate, which, in turn, is converted to nitrite by leaf surface microbes. We have previously shown that expression of a constitutively activated nitrate reductase (NR) enzyme dramatically decreases leaf nitrate levels in burley tobacco, resulting in substantial TSNA reductions without altering the alkaloid profile. Here, we show that plants expressing a constitutively active NR construct, designated 35S:S523D-NR, display an early-flowering phenotype that is also associated with a substantial reduction in plant biomass. We hypothesized that crossing 35S:S523D-NR tobaccos with burley cultivars that flower later than normal would help mitigate the undesirable early-flowering/reduced-biomass traits while maintaining the desirable low-nitrate/TSNA phenotype. To test this, 35S:S523D-NR plants were crossed with two late-flowering cultivars, NC 775 and NC 645WZ. In both cases, the plant biomass at harvest was restored to levels similar to those in the original cultivar used for transformation while the low-nitrate/TSNA trait was maintained. Interestingly, the mechanism by which yield was restored differed markedly between the two crosses. Biomass restoration in F_1_ hybrids using NC 645WZ as a parent was associated with delayed flowering, as originally hypothesized. Unexpectedly, however, crosses with NC 775 displayed enhanced biomass despite maintaining the early-flowering trait of the 35S:S523D-NR parent.

## Introduction

Tobacco smoke is composed of more than 7000 chemicals, out of which at least 69 have shown a possible association with carcinogenesis in animal and/or epidemiological studies^1,2^. Combustible tobacco products are considered more harmful than smokeless tobacco products, because the majority of the characterized toxicants found in tobacco smoke are the products of combustion. Tobacco-specific nitrosamines (TSNAs), however, are an exception, as a significant amount of TSNAs exist in the unburned tobacco filler. TSNAs are among the more notable of the harmful and potentially harmful chemicals found in tobacco smoke. TSNAs are formed when tobacco alkaloids—nitrogenous secondary metabolites produced in roots and stored within leaf vacuoles^3–5^—react with nitrosating agents^6^ . The specific TSNAs derived from nicotine and nornicotine, 4-(methylnitrosamino)-1-(3-pyridyl)-1-butanone (NNK) and *N-*nitrosonornicotine (NNN), respectively, are the most problematic, as they have been designated as group I carcinogens by the International Agency for Research on Cancer^7^.

Among the factors that can influence the amount of TSNAs found in tobacco cured leaf are nitrogen fertilization levels, growth environment, curing conditions, tobacco genotype, stalk position, and storage conditions^8,9^. Among the various market types used for producing cigarettes, burley tobaccos are the most challenging with respect to minimizing TSNA production and accumulation, for the following reasons: (1) the propensity of burley varieties to produce high levels of the NNN alkaloid precursor, nornicotine; (2) the high nitrate content of the leaf relative to other tobacco types; and (3) difficulties in controlling environmental conditions during the air-curing process commonly used for burley tobaccos immediately post-harvest. The first of these issues can be mitigated either through a screening process which has been implemented by the seed industry to eliminate high-nornicotine plants during seed scale-up^10^ or through introduction of genetic mutations that ensure minimal biosynthesis of nornicotine in the plant^11,12^. The high-nitrate-accumulating phenotype of burley tobaccos has been attributed to two homozygous recessive mutant alleles at the *Yellow Burley 1 (Yb1)* and *Yellow Burley 2 (Yb2)* loci, which largely define this market class. Tobacco plants homozygous for the *yb1* and *yb2* mutations are chlorophyll-deficient and impaired in their nitrogen utilization efficiency^13,14^. As a consequence, burley tobaccos require high levels of nitrogen fertilization and accumulate atypically large amounts of nitrate in their leaves. Although nitrate cannot directly interact with tobacco alkaloids to produce TSNAs, during the weeks-long air-curing period, leaf surface microbes can reduce nitrate (NO_3_^−^) to the highly reactive compound nitrite (NO_2_^−^), which can readily convert secondary alkaloids to TSNAs^9,15^.

We have previously shown that it is possible to reduce the levels of nitrate in air-cured burley tobaccos by introducing a constitutively activated nitrate reductase (NR) enzyme, leading to a substantial reduction in TSNA accumulation in cured leaves as well as in mainstream smoke^16^. Constitutive activation of the NR enzyme is achieved through substitution of a Ser residue at position 523 with the amino acid Asp. The S523D substitution prevents the NR enzyme from being phosphorylated and dephosphorylated at this position, which is an important regulatory mechanism which enables the enzyme to be active during the day and inactivated in the dark^17,18^. When the deregulated tobacco NR cDNA was placed downstream of the strong constitutive 35S promoter of the Cauliflower mosaic virus (a construct designated 35S:S523D-NR) and transformed into burley line DH98-325-6#775, the nitrate levels in the leaf were reduced by approximately 95%, and total TSNA levels were reduced by 77.5% in air-cured upper-stalk position leaves^16^. DH98-325-6#775 was selected for these studies because it possesses an ethyl methane sulfonate (EMS)-induced knockout mutation in *CYP82E4*, the unstable nicotine demethylase gene, whose variability in expression can lead to substantial plant-to-plant differences in the levels of the NNN precursor nornicotine and, thus, potentially confound TSNA analyses if not inactivated. Although DH98-325-6#775 was a good background for testing the effects of nitrate reduction technologies on TSNA reduction because it was a first-generation selection from a large EMS mutagenesis population^12^, the presence of a multitude of secondary EMS mutations scattered throughout its genome make it a poor background for establishing the effects of the 35S:S523D-NR transgene on other agronomic traits, such as yield.

In this study, we expand on our previous findings by introducing the 35S:S523D-NR construct into a superior, commercial-grade genetic background where effects of the transgene on agronomic properties could be assessed. Here we show in both growth chamber and field analyses that the transgene confers an early-flowering phenotype and causes a substantial reduction in overall plant biomass, traits that could preclude the consideration of this technology from practical application. In an attempt to mitigate the yield penalty associated with plants containing the 35S:S523D-NR transgene we crossed these lines to two late-flowering burley cultivars. The reduced biomass phenotype was largely alleviated in the resulting F_1_ hybrids while displaying no diminishment in the efficacy of the transgene to reduce TSNAs. The results presented here provide interesting insights into the physiological consequences of introducing a deregulated NR reductase transgene into burley tobacco plants. Furthermore, the information gained from this study reveals a means by which deployment of an effective harm reduction trait in tobacco could be realized without negatively impacting yield.

## Results

### Analysis of the effects of the deregulated NR transgene on aerial biomass

To determine whether strong expression of an NR enzyme whose activity could no longer be regulated via phosphorylation/dephosphorylation at the Ser residue at position 523 affected normal plant growth and development, it was important to transfer the transgene into a superior genetic background. TN90e4e5 is a near-isogenic derivative of the popular commercial variety TN90, which possesses the favorable agronomic characteristics of TN90 in addition to the stable low-nornicotine trait. The 35S:S523D-NR construct was introduced into TN90e4e5 by *Agrobacterium*-mediated transformation. Five T_0_ plants were selected for further investigation on the basis of their ultra-low-nitrate phenotypes and transgene expression levels, as determined by quantitative RT-PCR (Supplementary Fig. S1). These plants were designated GH3-1, GH5-2, GH5-5, GH8-1, and GH8-5. A vector-only control plant (GH10-4) was also included for comparison. To provide a preliminary assessment of the effect of the 35S:S523D-NR construct on normal plant growth and development, T_1_ plants from each of the five independent T_0_ events and the one vector-only T_0_ individual were evaluated in the field. Additional controls included TN90e4e5 wildtype plants and a line containing the 35S:S523D-NR transgene in the original DH98-325–6#775 background. Because the transgenic lines in TN90e4e5 were segregating for the transgene, PCR primers specific for the *nptII* selectable marker were used to screen young plants growing in float trays prior to transplantation to the field, in order to remove nontransgenic segregants. For line GH3-1, 279 of 379 progeny genotyped were positive for *nptII*; for GH8-5, 357 out of a total of 460 plants assayed were *nptII* positive. In both cases, the segregation patterns were consistent with a 3:1 ratio expected for a single locus event (χ_1_^2^ = 0.388, p = 0.533 for GH3-1; χ_1_^2^ = 1.670, p = 0.196 for GH8-5). In contrast, nearly all of the plants genotyped from float trays for GH5-2, GH5-5 and GH8-1 were *nptII* positive, suggesting multiple transgene insertions in these individuals. Half of the plants transplanted to the field were fertilized at a rate of 150 kg N/ha and the other half were provided 300 kg N/ha.

**Figure 1:**
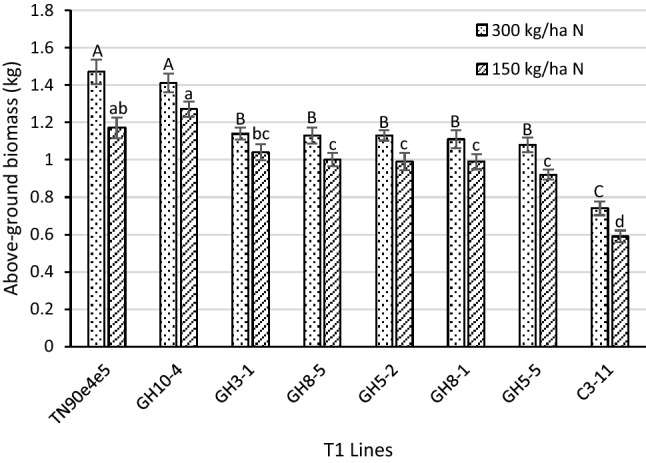
Above-ground biomass of T1 lines under two fertilization levels in 2014. Means ± standard errors of means with the same letter (upper case for the higher fertilization rate, lower case for the lower rate) are not significantly different at a 5% significance level according to REGWQ-based groupings. Sample sizes per line ranged from 29 to 39. TN90e4e5 = wild type; C3-11 = 35S:S523D-NR construct in DH98-325-6#775; GH10-4 = vector control in TN90e4e5; GH3-1, GH8-5, GH5-2, GH8-1, and GH5-5 = 35S:S523D-NR construct in TN90e4e5.

At harvest, each plant was cut at the base and weighed. As expected, the mean biomass for all genotypes was greater when the plants were fertilized with 300 kg N/ha than with 150 kg N/ha. As shown in Fig. 1, a significant reduction in above-ground biomass was observed in all lines that possessed the 35S:S523D-NR transgene at both fertilization levels (p < 0.0001). For transgenic plants in the TN90e4e5 background with the 35:S523D-NR transgene, the average reduction in biomass relative to the wildtype control was 16% at the 150 kg/ha N fertilization rate and 24% in plants fertilized with 300 kg/ha N. No significant differences were observed between the empty-vector control line GH10-4 and the wildtype TN90e4e5. The poorest yielding line was C3-11, which is the 35S:S523D-NR transgenic line in the original unimproved DH98-325-6#775 mutagenized background. Although recording time to flowering was not an objective of this study, as the plants began reaching maturity, it was informally observed that the lines with the 35S:S523D-NR transgene appeared to flower somewhat earlier than the wildtype and vector control genotypes.

### Growth and analysis of plants in a controlled environment

In order to gain a more complete understanding of the physiological consequences of maintaining low pools of free nitrate through constitutive expression of a deregulated NR enzyme, a line derived from the original GH8-5 event that had been fixed for the 35S:S523D-NR transgene was grown together with control plants in a controlled environmental chamber. Forty-eight plants of each genotype were grown under identical conditions through 8 weeks post-germination, at which point half of the plants of each genotype were watered under a medium (8 mM) nitrate fertilization regimen, while the other half were fertilized with a solution containing a higher (19 mM) concentration of nitrate. Sixteen days after initiation of the differential fertilization treatments, half of the plants of each genotype and fertilization level were measured for height and excised at the base to allow measurement of aerial biomass. Supplementary Fig. S2 shows this first cohort of plants on the day of data collection. The remaining plants were grown for an additional 2 months and 10 days with the same differential N fertilization treatments. Each of these plants was topped just prior to the first flower opening, and all suckers that emerged after topping were removed by hand until the end of the experiment. Supplementary Fig. S3 shows a representative subsample of the second cohort of plants, 1 day prior to the final harvest. Measurements taken on the second set of plants included flowering time (i.e., topping date), plant height, aerial biomass, and the number of harvestable leaves per plant (not counting those that were brown and shriveled at the base).

The results of the controlled environmental chamber study are shown in Tables 1 and 2. No genotype by fertilization interaction was observed; therefore, the data in Table 1 are presented as the effects of fertilization rate across the pooled averages of both genotypes. Not surprisingly, in both young and mature plants, the height and above-ground biomass were greater in plants fertilized with the higher nitrate level (although, the height difference in mature plants was not considered statistically significant; Table 1). The fertilization regime also affected flowering date and harvestable leaf number, as plant receiving 19 mM nitrate flowered on average 10 days earlier and had 1.7 fewer leaves than plants provided 8 mM nitrate.

**Table 1:** Effects of fertilization on burley tobacco height, biomass, flowering time and number of harvestable leaves.

Fertilization	Young plants (07/27/15)			Mature plants (10/07/15)				
	N	Height (cm)*	Above-soil biomass (g)	N	Average flowering date	Height (cm)**	Above-soil biomass (g)	Harvestable leaf #
19 mM NO_3_	24	9.8 ± 1.1^A^	180 ± 18.3^A^	24	Aug 26 ± 4.4 days^A^	89 ± 10.6^A^	814 ± 71.7^A^	21.8 ± 2.9^A^
8 mM NO_3_	24	8.3 ± 0.9^B^	121 ± 8.8^B^	22	Sept 5 ± 5.9 days^B^	86 ± 10.1^A^	617 ± 53.6^B^	23.5 ± 2.6^B^

Means ± standard deviations with the same letter are not significantly different at α = 0.05 according to REGWQ grouping.

*Height measured at apical meristem.

**Height measured at site of topping.

**Table 2:** Effect of the 35S:S523D-NR transgene on burley tobacco height, biomass, flowering time and number of harvestable leaves.

Genotype	Young plants (07/27/15)			Mature plants (10/07/15)				
	N	Height (cm)*	Above-soil biomass (g)	N	Average flowering date	Height (cm)**	Above-soil biomass (g)	Harvestable leaf #
35S:S523D-NR	24	9.2 ± 1.3^A^	148 ± 32.9^A^	22	Aug 27 ± 4.9 days^A^	83 ± 7.6^A^	686 ± 90^A^	20.5 ± 1.5^A^
TN90e4e5	24	8.9 ± 1.3^A^	153 ± 33.3^A^	24	Sept 4 ± 6.8 days^B^	91 ± 11.2^B^	751 ± 132^B^	24.5 ± 2.5^B^

Means ± standard deviations with the same letter are not significantly different at α = 0.05 according to REGWQ grouping.

*Height measured at apical meristem.

**Height measured at site of topping.

The data in Table 2 are presented as the effects of genotype across the pooled averages of both N-treatment levels. In young tobacco plants, there was no significant difference in plant height or above-ground biomass that could be attributed to the 35S:S523D-NR transgene. However, by the final harvest more than 2 months later, the 35S:S523D-NR plants were 8 cm shorter on average, had accumulated about 9% less above-ground biomass, and had four fewer leaves. Overall, plants that expressed the 35S:S523D-NR construct flowered an average of 8 days earlier than the control plants. The observation that the 35S:S523D-NR transgene was responsible for premature flowering and reduced leaf number, regardless of N-fertilization level, is readily apparent when the genotypic data is presented according to fertilization treatment. As shown in Supplementary Table S1, the transgenic line flowered on average seven days and nine days earlier than the TN90e4e5 control at the 19 mM and 8 mM nitrate treatment levels, respectively. Under both nitrate levels the 35S:S523D-NR plants produced approximately four fewer leaves. These experiments provided quantitative data supporting our informal field observations suggesting that the 35S:S523D-NR transgene confers an early-flowering phenotype. As flowering marks the developmental stage where photosynthates begin to be directed away from vegetative growth and toward reproductive tissues, premature flowering can lead to a reduction in the vegetative biomass of the plant. Additionally, in tobacco, the cultural practice of topping just prior to flowering may further contribute to yield inequities, as tobacco plants that flower prematurely tend to have fewer leaves at the time of topping.

### Crossing 35S:S523D-NR plants with late-flowering burley genotypes

Cumulatively, the results from the environmental chamber study and observations from an initial field experiment suggested that premature flowering may be the major, if not sole, factor responsible for the reduction in biomass associated with plants expressing the 35S:S523D-NR transgene. If true, we hypothesized that crossing 35S:S523D-NR lines with burley cultivars that display uncharacteristically late-flowering phenotypes may at least partially alleviate the early-flowering, reduced-yield phenotypes associated with the transgene. Among burley tobacco parental lines maintained by the North Carolina State University (NCSU) tobacco breeding program, lines NC775 and NC645WZ are known to flower much later than most genotypes of this market type (Ramsey Lewis, personal observation). Furthermore, in the preliminary field trial conducted in 2014 (Fig. 1), all plants derived from the same T_0_ parent displayed uniformly low levels of nitrate (data not shown), despite the fact that these were T_1_ plants still segregating for the transgene (wildtype segregants were removed after genotyping). This suggested that a single copy of the transgene may be as effective as two copies in reducing the nitrate content of the leaf. Two independent 35S:S523D-NR lines that were fixed for the transgene, GH3-1 and GH8-5, were crossed individually to NC775 and NC645WZ. The resulting F_1_ hybrids, along with appropriate controls, were evaluated in the field.

### Analysis of 35S:S523D-NR plants crossed with late-flowering burley lines

#### Flowering time

We conducted two successive field trials in 2017 and 2018 with the parental lines GH3-1, GH8-5, NC645WZ, and NC775; the four corresponding F1 hybrids, GH3-1 ×  NC775, GH3-l  ×  NC645WZ, GH8-5 ×  NC775, and GH8-5 ×  NC645WZ; and TN90e4e5 (the wildtype background of the GH3-1 and GH8-5 transgenic lines). Table 3 shows the average flowering dates of the parental lines, their F1 hybrids, and the wildtype TN90e4e5 control for both years. Because of an approaching hurricane that year, the 2018 experiment was terminated earlier than the 2017 experiment. At the time of the emergency harvest in 2018, a considerable proportion of the plants had not yet flowered, especially in lines NC645WZ, NC775, GH8-5 × NC645WZ, GH3-1 × NC645WZ, and TN90e4e5 (Table 3). The 2017 experiment also had some plants that had not flowered by the time of harvest, especially in case of the late-flowering parents NC645WZ and NC775. To address this problem, the flowering dates were used to calculate the number of days from transplanting to flowering, which was used in a time-to-event analysis. Plants that had not flowered by harvest were considered to have censored time to flowering. An overall analysis and pairwise comparison of the time to flowering of the nine lines in each year were performed by using a log-rank test for each year. The results of multiple comparisons were summarized by using groupings with Bonferroni correction.

**Table 3:** Flowering dates of the 35S:S523D-NR lines, their F1 hybrids with late-flowering burley cultivars, and parental controls in 2017 and 2018.

Line	2018				2017			
	Percent of plants flowered by harvest	Number of plants flowered by harvest	Average of available	Grouping with Bonferroni correction	Percent of plants flowered by harvest	Number of plants flowered by harvest	Average of available	Grouping with Bonferroni correction
			Flowering dates				Flowering dates	
NC645WZ	1.6	1	Aug-24	A	10.7	3	Sep 15 ± 4.3 d	A
NC775	53.8	35	Aug 24 ± 1.2 d	B	67.6	23	Aug 25 ± 1.6 d	B
GH8-5 × NC645WZ	68.3	41	Aug 25 ± 1.5 d	BC	100	32	Aug 18 ± 1.4 d	C
GH3-1 × NC645WZ	81	51	Aug 22 ± 1.2 d	C	93.8	30	Aug 14 ± 1.7 d	C
TN90e4e5	75.4	46	Aug 19 ± 1.2 d	CD	93.5	29	Aug 15 ± 1.8 d	C
GH8-5 × NC775	89.4	59	Aug 15 ± 1.0 d	DE	94.1	32	Aug 6 ± 2.2 d	CD
GH8-5	92.1	58	Aug 16 ± 1.2 d	EF	100	27	Aug 4 ± 1.3 d	D
GH3-1 × NC775	93.4	57	Aug 14 ± 1.1 d	EF	96.9	31	Aug 4 ± 2.2 d	CD
GH3-1	97	64	Aug 10 ± 1.1 d	F	100	22	Aug 6 ± 2.3 d	CD

Pairwise comparisons of days from transplanting to flowering or harvest were performed by using the log-rank test.

Lines with the same letter are not significantly different in time from transplanting to flowering at a 5% significance level with Bonferroni correction.

Available flowering dates of each line were summarized in means ± standard errors of means; d, days.

In 2018, the days to flowering of GH8-5 × NC645WZ and GH3-1 × NC645WZ were significantly longer than those of GH8-5 and GH3-1, respectively (Table 2), but not significantly different from that of TN90e4e5. This suggests that crossing to NC645WZ significantly delayed the flowering of GH8-5 and GH3-1 and, thereby, extended the average flowering date of both lines to a degree similar to that of their wildtype parent TN90e4e5. The average delays were about 9 days and 12 days for lines GH8-5 and GH3-1, respectively. Interestingly, the days to flowering of the GH8-5 × NC775 and GH3-1 × NC775 hybrids were not significantly different from those of GH8-5 and GH3-1, respectively, indicating that crossing to NC775 was not effective in delaying the flowering time of the transgenic lines GH8-5 and GH3-1.

In 2017, the GH8-5 × NC645WZ plants flowered, on average, 14 days later than the GH8-5 individuals. The delay was statistically significant. However, under Bonferroni correction, the GH3-1 × NC645WZ hybrids were not significantly different from GH3-1 in terms of time to flowering. For pairwise comparisons of the nine lines, as in this case, a Bonferroni correction requires a critical p value of ≤ 0.0014 to declare significance at a 5% significance level, which is extremely conservative. An independent comparison of GH3-1 × NC645WZ with GH3-1 generated a p value of 0.0069, which is highly significant without Bonferroni correction. Furthermore, the actual extent of the delay in flowering was about 8 days, which is consistent with the degree to which crossing the 35S:S523D-NR transgenic lines to NC645WZ delayed flowering in 2018. Cumulatively, the results from both the 2017 and 2018 field seasons strongly support the conclusion that crossing GH3-1 and GH8-5 with NC645WZ results in F_1_ hybrids that display flowering-time phenotypes similar to that of TN90e4e5. In contrast, in both the 2017 and 2018 field seasons, GH8-5 × NC775 and GH3-1 × NC775 hybrids were not significantly different from their GH8-5 and GH3-1 parental lines, respectively, in days to flowering.

#### Biomass

**Figure 2:**
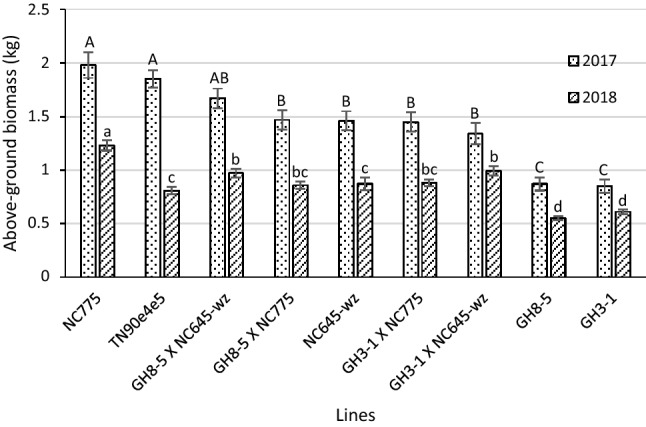
Above-ground biomass of F1 hybrids between the 35S:S523D-NR lines and late-flowering breeding lines and their parents, grown at Clayton in 2017 and 2018. Means ± standard errors of means with the same letter are not significantly different at a 5% significance level according to REGWQ groupings. Sample sizes per line ranged from 26 to 34 in 2017 and 58 to 64 in 2018.

The above-ground biomass of F1 hybrids between the 35S:S523D-NR lines and late-flowering burley cultivars is shown in Fig. 2. Overall, plant growth and vigor were superior in the 2017 field season than in 2018. In both years, the homozygous transgenic parental lines GH3-1 and GH8-5 displayed the lowest average biomass, while NC775 had accumulated the greatest biomass by the time of harvest. All hybrids between the 35S:S523D-NR lines and late-flowering burley cultivars accumulated significantly greater amounts of biomass than the GH3-1 and GH8-5 parents in both 2017 and 2018. For crosses with NC645WZ, these results are not surprising because of the delayed flowering response observed in these hybrids (Table 3). The observation that crossing GH3-1 and GH8-5 with NC775 also significantly enhanced biomass accumulation was unexpected, given that the flowering phenotype in these hybrids did not differ significantly from that of the 35S:S523D-NR parent.

The degree to which crossing the 35S:S523-NR lines to late-flowering burley types restored plant biomass to levels similar to that of TN90e4e5 appeared to vary according to year/environment. In 2017, when the environmental conditions were favorable, TN90e4e5 outperformed all four hybrids (though, in comparison to the GH8-5 × NC645WZ hybrid, the increase was not considered statistically significant). In 2018, however, the average biomass for all four hybrid combinations was numerically greater than that of TN90e4e5. In two cases—the GH8-5 × NC645WZ and GH3-1 × NC645WZ hybrids—the increases in biomass were considered statistically significant (Fig. 2).

### Nitrate, TSNA, and alkaloid data from air-cured leaves

**Figure 3:**
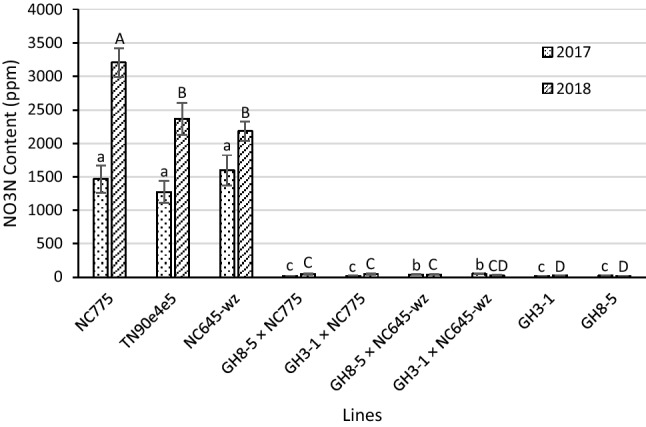
Nitrate (NO3N) content in cured leaves of F1 hybrids between the 35S:S523D-NR lines and late-flowering breeding lines and their parents, grown in 2017 and 2018. Means ± standard errors of means with the same letter are not significantly different at a 5% significance level according to REGWQ groupings. Sample sizes per line ranged from 22 to 33 in 2017 and 58 to 64 in 2018.

One of the primary objectives of this research was to establish whether it would be possible to deploy the 35S:S523D-NR-mediated low-nitrate, low-TSNA technology in a manner that would not lead to a severe reduction in yield. The results reported above suggest that the problems associated with flowering time and/or yield when the transgene was introduced into TN90e4e5 could largely be overcome through generation of F1 hybrids involving the late-flowering burley lines. Equally important, however, was determining whether the hybrid materials retained their low-nitrate/-TSNA properties. The nitrate contents of the materials that were field tested in 2017 and 2018 are shown in Fig. 3 and Tables 4 and 5. During a given year, the nitrate levels of the three lines included in this study that lacked the 35S:S523D-NR transgene—that is, NC775, NC645WZ, and TN90e4e5—were similar, with the exception of NC775 in 2018, which displayed significantly higher nitrate content than the other two wildtype lines that year. In contrast, all materials possessing the 35S:S523D-NR construct, whether homozygous (GH3-1 and GH8-5) or hemizygous (the hybrids), displayed extreme reductions in nitrate levels in the cured leaf, ranging from 1.7% to 4.4% in 2017 (Table 4) and 1.4% to 2.2% in 2018 (Table 5) of the proportions observed in the TN90e4e5 control background.

**Table 4:** Nitrate, TSNA, and alkaloid contents of F1 hybrids between 35S:S523D-NR lines and late flowering cultivars in 2017.

Line	TN90e4e5	NC775	NC645WZ	GH3-1	GH8-5	GH3-1 × NC775	GH3-1 × NC645WZ	GH8-5 × NC775	GH8-5 × NC645WZ
NO3N (ppm)	1274 ± 160.5^A^	1467 ± 201.3^A^	1596 ± 226.9^A^	23 ± 2.2^C^	28 ± 5.5^C^	25 ± 2.6^C^	56 ± 18.3^B^	22 ± 2.3^C^	44 ± 4.4^B^
NNN (ng/g)	421 ± 99^A^	176 ± 32^B^	133 ± 12^B^	89 ± 6^C^	89 ± 4^C^	87 ± 5^C^	87 ± 4^C^	84 ± 5^C^	92 ± 6^C^
NNK (ng/g)	98 ± 12.1^A^	82 ± 13.5^A^	96 ± 12.1^A^	39 ± 3.5^B^	41 ± 3.1^B^	33 ± 2.3^B^	39 ± 3.8^B^	33 ± 3.6^B^	44 ± 5.0^B^
NAT (ng/g)	566 ± 181^A^	302 ± 93^B^	163 ± 29^B^	110 ± 12^B^	118 ± 10^B^	95 ± 7^B^	90 ± 6^B^	99 ± 13^B^	96 ± 8^B^
NAB (ng/g)	25 ± 4.36^A^	31 ± 8.10^A^	24 ± 5.89^A^	15 ± 3.19^AB^	12 ± 1.24^AB^	13 ± 2.73^B^	11 ± 1.20^AB^	9 ± 0.93^B^	12 ± 1.69^AB^
Total TSNA (ng/g)	1110 ± 291^A^	591 ± 142^B^	374 ± 63^B^	252 ± 18^C^	259 ± 15^C^	227 ± 12^C^	227 ± 11^C^	225 ± 20^C^	244 ± 19^C^
Nicotine (%)	3.4 ± 0.31^AB^	2.1 ± 0.30^CD^	1.5 ± 0.19^D^	4.1 ± 0.29^AB^	4.3 ± 0.24^A^	3.8 ± 0.27^AB^	3.0 ± 0.30^BC^	3.5 ± 0.30^AB^	3.1 ± 0.31^ABC^
Nornicotine (%)	0.066 ± 0.0062^AB^	0.021 ± 0.0023^C^	0.017 ± 0.0012^C^	0.078 ± 0.0077^AB^	0.085 ± 0.0063^A^	0.069 ± 0.0072^AB^	0.055 ± 0.0068^B^	0.058 ± 0.0062^B^	0.056 ± 0.0066^B^
Anabasine (%)	0.012 ± 0.0012^BCD^	0.008 ± 0.0011^DE^	0.005 ± 0.0006^E^	0.017 ± 0.0016^AB^	0.019 ± 0.0014^A^	0.015 ± 0.0012^ABC^	0.011 ± 0.0014^CD^	0.014 ± 0.0013^BC^	0.013 ± 0.0016^BCD^
Anatabine (%)	0.101 ± 0.013^C^	0.052 ± 0.009^D^	0.036 ± 0.004^D^	0.157 ± 0.014^AB^	0.181 ± 0.014^A^	0.131 ± 0.011^BC^	0.097 ± 0.015^C^	0.115 ± 0.012^BC^	0.103 ± 0.013^C^
TA (%)	3.6 ± 0.33^AB^	2.2 ± 0.31^CD^	1.5 ± 0.19^D^	4.3 ± 0.31^AB^	4.6 ± 0.26^A^	4.0 ± 0.28^AB^	3.2 ± 0.32^BC^	3.7 ± 0.31^AB^	3.3 ± 0.33^ABC^

Means ± standard errors of means with the same letter are not significantly different at 5% significance level according to REGWQ grouping.

Sample sizes per line ranged from 22 to 33 for NO3N and 9 to 10 for TSNA and alkaloids.

**Table 5:** Nitrate, TSNA, and alkaloid contents of F1 hybrids between 35S:S523D-NR lines and late flowering cultivars in 2018.

Line	TN90e4e5	NC775	NC645WZ	GH3-1	GH8-5	GH3-1 × NC775	GH3-1 × NC645WZ	GH8-5 × NC775	GH8-5 × NC645WZ
NO3N (ppm)	2366.5 ± 238.2^B^	3207.9 ± 212.6^A^	2182.8 ± 143.8^B^	27.3 ± 3.7^D^	23.4 ± 1.8^D^	48.4 ± 12.0^C^	33.7 ± 4.3^CD^	51.4 ± 8.0^C^	45.3 ± 5.4^C^
NNN (ng/g)	317.2 ± 63.9^A^	125.2 ± 8.3^B^	98.3 ± 6.2^BC^	95.8 ± 8.1^BC^	91.6 ± 6.7^BC^	90.2 ± 8.7^C^	66.5 ± 3.6^D^	93.6 ± 5.2^BC^	71.4 ± 8.9^D^
NNK (ng/g)	89.4 ± 9.5^A^	65.0 ± 6.2^B^	58.7 ± 3.0^B^	40.0 ± 2.9^C^	36.5 ± 3.1^C^	38.9 ± 3.6^C^	29.6 ± 1.9^C^	34.5 ± 2.9^C^	30.5 ± 3.3^C^
NAT (ng/g)	273.0 ± 54.6^A^	189.2 ± 28.4^A^	146.0 ± 12.0^AB^	107.9 ± 10.7^BC^	107.0 ± 7.7^BC^	95.8 ± 6.6^CD^	75.2 ± 4.1^D^	103.8 ± 6.3^BC^	82.0 ± 14.1^D^
Total TSNA (ng/g)	680 ± 124.7^A^	379 ± 40.4^AB^	303 ± 19.6^BC^	244 ± 20.8^C^	235 ± 17.0^C^	225 ± 17.7^C^	171 ± 8.6^D^	232 ± 13.5^C^	184 ± 25.8^D^
Nicotine (%)	3.0 ± 0.32^BC^	2.5 ± 0.23^C^	2.1 ± 0.17^C^	3.9 ± 0.31^AB^	4.4 ± 0.23^A^	3.3 ± 0.37^ABC^	2.5 ± 0.31^C^	3.7 ± 0.20^AB^	2.6 ± 0.35^C^
Nornicotine (%)	0.051 ± 0.0054^A^	0.015 ± 0.0016^C^	0.012 ± 0.0012^C^	0.056 ± 0.0070^A^	0.064 ± 0.0052^A^	0.047 ± 0.0054^AB^	0.032 ± 0.0040^B^	0.050 ± 0.0035^A^	0.033 ± 0.0055^B^
Anabasine (%)	0.0084 ± 0.00109^BCD^	0.0063 ± 0.00100^CD^	0.0052 ± 0.00055^D^	0.0122 ± 0.00219^AB^	0.0151 ± 0.00127^A^	0.0112 ± 0.00143^ABC^	0.0072 ± 0.00132^BCD^	0.0127 ± 0.00094^AB^	0.0076 ± 0.00161^BCD^
Anatabine (%)	0.057 ± 0.0086^CDE^	0.038 ± 0.0065^E^	0.036 ± 0.0033^E^	0.112 ± 0.0141^AB^	0.120 ± 0.0111^A^	0.078 ± 0.0107^BCD^	0.053 ± 0.0102^DE^	0.089 ± 0.0078^ABC^	0.056 ± 0.0137^CDE^
TA (%)	3.1 ± 0.33^BCD^	2.6 ± 0.24^CD^	2.2 ± 0.18^D^	4.1 ± 0.33^AB^	4.6 ± 0.24^A^	3.4 ± 0.38^ABC^	2.6 ± 0.33^CD^	3.9 ± 0.21^AB^	2.7 ± 0.37^CD^

Means ± standard errors of means with the same letter are not significantly different at 5% significance level according to REGWQ grouping.

Sample sizes per line ranged from 58 to 64 for NO3N and were 10 for all other traits.

**Figure 4:**
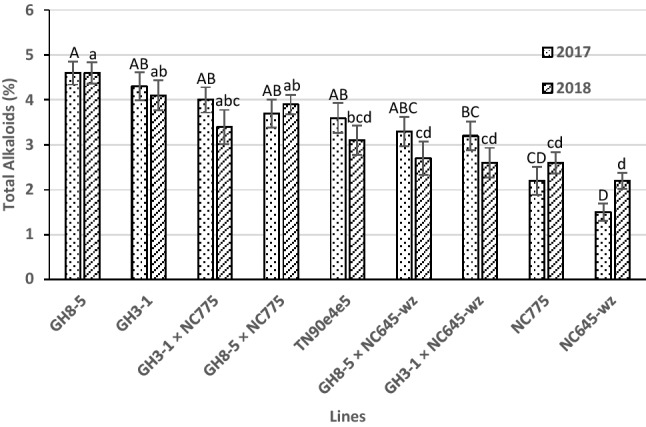
Total alkaloid content (% dry weight) in the cured leaves of F1 hybrids between the 35S:S523D-NR lines and late-flowering breeding lines and their parents grown in 2017 and 2018. Means ± standard errors of means with the same letter are not significantly different at a 5% significance level according to REGWQ groupings. Sample sizes per line ranged from 9 to 10 in 2017 and were 10 in 2018.

In addition to the nitrate content of the leaf, there is also a direct positive correlation between alkaloid content and TSNAs in air-cured tobaccos. In order to assess the degree by which TSNA levels can be lowered through reducing nitrate accumulation, it is important that the results be interpreted within the context of differences in the alkaloid contents of the various lines tested. Figure 4 shows the total alkaloid concentrations in 2017 and 2018 of the nine genotypes included in this study. In both years, the total alkaloid levels were ~ 2-fold higher in the GH3-1 and GH8-5 parental lines than in NC775 and NC645WZ. In addition to being significant at the level of total alkaloids, each of the four individual alkaloids measured (nicotine, nornicotine, anatabine, and anabasine) were significantly higher in GH3-1 and GH8-5 than in NC775 and NC645WZ in both years (Tables 4 and 5). The reason for the higher alkaloid content in the 35S:S523D-NR parents than in the wildtype NC775 and NC645WZ cultivars is more likely attributable to the differences in when the plants were topped rather than any inherent genetic differences. The cultural practice of topping (followed by application of a sucker control agent to prevent the establishment of a new apical meristem) in tobacco serves two purposes: (1) It upregulates the genetic pathway responsible for alkaloid production, leading to an increase in nicotine and other pyridine alkaloid levels, and (2) it maximizes the flux of photosynthates into leaf expansion rather than towards production of floral tissues and seeds. Numerous NC775 and NC645WZ plants had yet to flower by the time of harvest and, thus, were never topped, and those plants that had been topped averaged a much shorter post-topping duration than the GH3-1 and GH8-5 individuals. Therefore, it is not surprising that the 35S:S523D-NR parents accumulated higher levels of alkaloids by harvest. Further supporting this conclusion is the observation that the hybrid lines and TN90e4e5, which averaged intermediate flowering times and, thus, post-topping durations, also averaged intermediate levels of alkaloid accumulation (Fig. 4).

**Figure 5:**
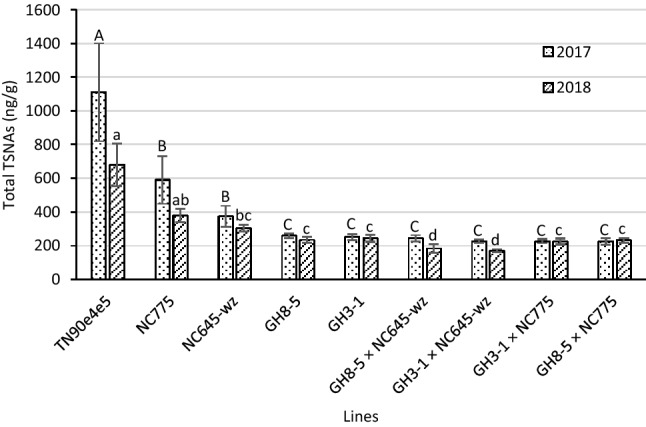
Total TSNA content in the cured leaves of F1 hybrids between the 35S:S523D-NR lines and late-flowering breeding lines and their parents, grown in 2017 and 2018. Means ± standard errors of means with the same letter are not significantly different at a 5% significance level according to REGWQ groupings. Sample sizes per line ranged from 9 to 10 in 2017 and were 10 in 2018.

Of the three wildtype lines included in this study, TN90e4e5 displayed the highest levels of total TSNAs in both 2017 and 2018 (Fig. 5). As these three lines accumulated similar levels of nitrate in a given year (Fig. 3), it is likely that the higher alkaloid content of TN90e4e5 drove greater TSNA production in this background relative to NC775 and NC645WZ. Because both nitrate and alkaloid levels greatly influence the propensity for TSNA formation, the best way to assess the effect of the nitrate-lowering phenotype mediated by the 35S:S523D-NR transgene on TSNAs is to compare materials that are the most similar in terms of alkaloid concentration, flowering time, and plant biomass. Across both years, in all three of these parameters, hybrids GH3-1 × NC645WZ and GH8-5 × NC645WZ were the most similar to the TN90e4e5 genotype used to generate GH3-1 and GH8-5 (Fig. 2; Tables 3–5). In 2017, these two hybrid combinations showed average reductions in NNN, *N-*nitrosoanatabine (NAT), *N-*nitrosoanabasine (NAB), and NNK of ~ 79%, ~ 83%, ~ 54%, and ~ 58%, respectively, relative to the TN90e4e5 plants (Table 4). The results obtained in 2018 were very similar. That year, the NNN, NAT, and NNK reductions averaged ~ 78%, ~ 71%, and ~ 66%, respectively, in the two 35S:S523D-NR lines crossed to NC645WZ, relative to TN90e4e5 (Table 5).

## Discussion

We have previously demonstrated that constitutive expression of an NR variant that has lost the ability to be regulated via reversible phosphorylation (S523D-NR) was effective in dramatically lowering the nitrate levels in the leaves of burley tobacco plants^16^. Consistent with previous reports^13,15,19^, the reduced-nitrate phenotype in these plants was associated with significant reductions in all classes of TSNAs. In order for any novel trait to be commercially deployed in a crop species, however, it must first be demonstrated that other important agronomic characteristics are not impacted to an unacceptable degree. Initial field evaluation of the 35S:S523D-NR lines suggested that two major agronomic traits, flowering time and aerial biomass, were affected by the transgene. In a controlled environment growth study, plants possessing the 35S:S523D-NR transgene flowered, on average, 8 days earlier than the wildtype control. Similar results were observed in 2 years of field evaluation.

Regulation of flowering time in plants is complex, controlled by multiple environmental cues and signal transduction pathways^20^. Changes in nitrate availability and/or levels of N assimilates are among the factors known to influence flowering^21–23^. In some plant species, limitations in nitrate have been shown to trigger premature flowering^24–26^. Consistent with this, in our controlled environmental chamber study we observed that plants fertilized with 8 mM nitrate flowered approximately 10 days earlier than those treated with 19 mM nitrate, regardless of genotype (Table 1; Supplementary Table S1). Conversely, excess nitrate content in some studies has been shown to delay flowering^23,27^. The mechanisms by which changes in nitrate levels promote or delay flowering are still unclear. A study by Liu et al.^27^ found that low-nitrate stress in *Arabidopsis* activates gibberellic acid (GA) biosynthesis by increasing expression of the GA biosynthetic gene *GA1*, coupled with enhanced expression of the well-characterized floral inducers *CONSTANS (CO)* and *SUPPRESSOR OF OVEREXPRESSION OF CO1* (*SOC1)*. In a separate *Arabidopsis*-based study, however, it was concluded that limiting nitrate levels accelerated flowering in a GA-independent manner via a pathway that functions in the absence of previously characterized floral integrators, including CO and SOC1^24^.

Although the specific signaling effector(s) in nitrate-associated regulation of flowering is unknown, it is possible that the NR enzyme itself plays a direct role. In *Arabidopsis*, the protein phosphatase 2A regulatory subunit B55 (both α and β homologs) directly interacts with the NR enzyme and may function to dephosphorylate the regulatory Ser residue of NR at position 523^28^. These same B55 subunits have also been shown to be regulators of flowering time^29^. Furthermore, the NR enzyme is responsible for synthesis of nitric oxide (NO) from nitrite^30^. Among its numerous signaling functions, NO has been implicated in regulation of flowering^31^. NO represses flowering, and mutant plants deficient in NO production flower prematurely^32^. In addition, *Arabidopsis* plants possessing mutant alleles of the genes encoding NR emit less NO and blossom prematurely^33^. Interestingly, the results of a detached leaf assay in a previous study showed that *Nicotiana plumbaginifolia* plants expressing the same deregulated S523D-NR gene used in our study emitted significantly higher levels of NO in the dark than control plants^34^. Flowering time was not addressed in that report, however.

There is a well-established association in plants among flowering time, leaf number, and biomass yield^35–38^ (Salehi et al. 2005; Ni et al. 2008; Jensen et al. 2012; Murphy et al. 2014). In tobacco, these associations are further impacted by the agronomic practice referred to as topping. Under standard production practices, the uppermost part of the plant is removed once signs of floral development are observed. In addition to the apical meristem, topping also leads to the removal of several of the upper young leaves. Subsequent application of a suckercide prevents the establishment of a new apical/floral meristem and fixes the number of leaves at harvest to those that were present on the plant at the time of topping. Enhanced expansion of the leaves that remain after topping, however, is another characteristic of this practice, as photosynthates continue to be directed toward vegetative rather than reproductive growth. Thus, if a plant flowers prematurely, there will be fewer leaves at harvest, which can negatively impact yield. Indeed, in the environmental chamber study comparing the 35S:S523D-NR line GH8-5 with its TN90e4e5 control (which included topping just prior to the opening of the first flower), the early-flowering transgenic plants averaged four fewer leaves per plant at the end of the experiment (Table 2). Conversely, if a tobacco plant flowers abnormally late, the end biomass can also be reduced, as the plants will experience a shortened period of post-topping vegetative leaf expansion, assuming all materials are harvested at the same time (as was the case in both our growth chamber and field studies).

We speculated that premature flowering was the primary cause of the biomass reductions observed in 35S:S523D-NR lines relative to the TN90e4e5 background line in which the transgenics were produced. Though there is a general lack of understanding on why some tobacco genotypes flower at different times than others, we tested the possibility that making genetic crosses between the early-flowering 35S:S523D-NR lines and two burley parental inbred lines known to flower especially late could at least partially remediate the early-flowering/reduced-biomass phenotypes while retaining the desired low-nitrate/low-TSNA profiles. Crosses to NC645WZ appeared to validate this strategy. During both field seasons, the flowering time and average biomass of the hybrids more closely resembled those of the wildtype TN90e4e5 than the 35S:S523D-NR parental types. Importantly, the 35S:S523D-NR transgene was just as effective in reducing TSNA levels when present as a single copy in the hybrids, as it was in the GH3-1 and GH8-5 parents homozygous for two copies.

More surprising were the results observed when NC775 was used as the late-flowering parent in the hybrid crosses. Similar to crosses with NC645WZ, the biomass of the hybrids was significantly greater than that of the GH3-1 or GH8-5 parents. Unexpectedly, the average time to flowering was not statistically different among the NC775 crosses to the respective 35S:S523D-NR parent. There are two plausible explanations for the observed increase in biomass in the absence of time-to-flowering differences: (1) The average number of leaves per plant was greater in the hybrids at the time of topping and/or (2) the average leaf size, or capacity to expand post-topping, was greater in the hybrids. Leaf number and size were not recorded in 2017 or 2018—such measurements in future studies could help resolve this issue. Nevertheless, the results from this study strongly suggest that there are genetic determinants in NC645WZ that influence flowering time in a dominant or additive manner that are not present in NC775. Indeed, NC645WZ carries an introgressed chromosome segment derived from *N. rustica*, designated as ‘*Wz*’^39^, which seems to be associated with later flowering in many genetic backgrounds (Ramsey Lewis, personal observations).

In conclusion, early-flowering and reduced-biomass phenotypes were observed in burley tobacco plants that constitutively expressed an NR enzyme with an amino acid substitution that results in the enzyme being in a continuously activated state. The reduced-yield phenotype, in particular, could serve as a barrier to the consideration of this TSNA-reducing technology for commercial application. The results presented here show that by making crosses to burley plants that display atypically late-flowering phenotypes, the aerial biomass can at least in part be restored to a level similar to that of the genetic background of the line used for transformation of the 35S:S523D-NR construct. Interestingly, it appeared that yield enhancement in the F_1_ hybrid lines could be achieved through a flowering time-dependent or -independent mechanism, depending on which nontransgenic parent was used in the cross. Importantly, the efficacy of TSNA reduction mediated by the transgene was not diminished in the hybrids. Given the current practice of releasing new commercial tobacco varieties as F_1_ hybrids, it would be feasible to deploy a trait of this nature within existing schemes of tobacco variety development. Finally, the plant materials developed herein could help elucidate the genetic factors and mechanisms associated with flowering, biomass, and nitrate assimilation in tobacco in future investigations.

## Methods

### Transgenic plant generation and qRT-PCR analysis

Burley cultivar TN90e4e5 was developed by backcrossing the EMS-induced knockout mutation in *CYP82E4* found in line DH98-325-6#775 with an EMS-generated knockout mutation in the minor nicotine demethylase gene *CYP82E5*^12^ and then backcrossing both mutations seven times into the commercial burley variety TN90 LC^40^. The final backcross was followed by two rounds of self-fertilization to fix the *cyp82e4* and *cyp82e5* gene mutations to homozygosity (this line is referred to as NCBEX1F in the cited patent). TN90e4e5 was transformed with the previously described 35S:S523D-NR construct^16^ by using the leaf disc method^41^. The first fully expanded leaf from the top of each young T_0_ plants was detached at the petiole and treated with ethephon to induce senescence as described in Lu et al.^16^. Total RNA was isolated from half of the leaf using the Quick-RNA MiniPrep Kit (Zymo Research). The other leaf half was used for nitrate analysis. First strand cDNA was synthesized using Superscript II Reverse Transcriptase (Invitrogen). Primers NRq9F (5′-GTACTTCGAGTTGGTTGTCAA-3′) and NRq7R (5′-CTGTTTGCCATGAACTAAGAA-3′) were used amplify NR expression. Ethephon-treated materials were used to minimize accumulation of endogenous NR gene expression as these primers could not distinguish endogenous transcripts from those mediated by the 35S:S523D-NR construct. The primers EF1aqRTF-2 (5′-AGATGCACCACGAAGCTC-3′) and EF1aqRTR-2 (5′-CAATCTGTCCTGAATGGTTC-3′) were used to amplify the expression of the *EF-1α* gene, which is an endogenous house-keeping gene that served as an internal reference to normalize the expression of the transgene. PCR products were detected using the SYBR Green method (iTaq Universal SYBR Green Supermix, Bio-Rad, Hercules, CA). PCR reaction, product detection, and quantitation were performed using the Mx3005P qPCR System (Agilent Technologies, Santa Clara, CA). PCR reactions were carried out using the SYBR Green method (iTaq Universal SYBR Green Supermix, Bio-Rad, Hercules, CA) according to the manufacturer-recommended method. Three replicates were performed for each sample. The “Comparative Quantitation” module of the MxPro Software (the operational and analysis software for the Mx3005P qPCR instrument) was used for product quantitation and analysis. The module utilized the ΔΔC_t_ algorithm for relative quantitation. In this study, the expression level of the NR gene in each sample was expressed as a ratio of the quantity of the NR gene transcript to that of the *EF-1α* reference gene transcript (${2}^{-\Delta {C}_{t}}={2}^{-({C}_{{t}_{NR}}-{C}_{{t}_{EF-1\alpha }})})$.

### 2014 field study

T_1_ seeds from the low nitrate, high transgene expressing plants GH3-1, GH5-2, GH5-5, GH8-1 and GH8-5, along with an empty-vector control line (GH10-4), were seeded in float trays. The wildtype cultivar TN90e4e5 was used as a control. As a final control, line C3-11, a fixed 35S:S523D-NR transgenic line derived from the unimproved mutation event DH98-325–6#775^16^, was also included in this study. To eliminate nontrangenic segregants, the T_1_ transgenic lines GH3-1, GH5-2, GH5-5, GH8-1, and GH8-5 were genotyped by PCR by using primers specific for the *nptII* selectable marker gene. Transgene-positive seedlings were transplanted to a field environment near Clayton, NC, by using a randomized complete block design consisting of 80 replications with individual plants serving as experimental units. While 40 replications were fertilized with 150 kg N/ha, the remaining 40 replications were provided with 300 kg N/ha. The plants were otherwise maintained in accordance with recommended practices for tobacco production in North Carolina. Apical inflorescences were removed as the plants flowered, and development of apical meristems (suckers) was controlled by downstalk application of FluPro^®^ in accordance with the manufacturer’s instruction (Chemtura Agro Solutions, USA) and suggested rates. At harvest, each plant was cut at the base by using a machete and weighed to determine total aerial biomass.

### Controlled environmental chamber study

T_2_ generation plants from the fixed 35S:S523D-NR transgenic line GH8-5 and the corresponding TN90e4e5 control were grown in a controlled environmental chamber at the NCSU Phytotron (https://phytotron.ncsu.edu). Seedlings were grown on a 2:1 peat:sand mix for 7 weeks, at which time 48 GH8-5 and 48 TN90e4e5 plants were transferred to 6-in. pots filled with river sand. The pots were arranged in a randomized complete block design with individual plants representing experimental units. To allow adjustment for transplant shock, all plants were watered with a standard Phytotron nutrient solution for 8 days, followed by a differential fertilization regimen, where half of the plants were watered with an 8 mM nitrate solution and the other half with a 19 mM nitrate medium. After 16 days of differential fertilization treatment, half of the plants were harvested (young plant stage), at which time their height was recorded and the fresh weight of the aerial portion was measured after excision at the base. The remaining 48 plants continued to be fertilized with the 8 mM and 19 mM nitrate solutions for an additional 2.5 months. For this set of plants, topping was performed just prior to the opening of the first flower, and suckers were checked for and removed manually until the final harvest, when the plant height, biomass, and leaf number data were recorded. A complete description of the nutrient formulas for the 8 mM and 19 mM nitrate media as well as the lighting and temperature conditions can be found in Lu et al.^16^.

### Hybrid crosses and 2017 and 2018 field experiments

T_2_ transgenic lines GH3-1 and GH8-5, fixed for the 35S:S523D-NR transgene, were crossed with late-flowering burley inbred parental lines NC645WZ and NC775 to produce four F1 hybrids: GH3-1 × NC775, GH3-1 × NC645-wz, GH8-5 × NC775, and GH8-5 × NC645-wz. The four F1 hybrids and their four corresponding parental lines, together with TN90e4e5 (the background line used to develop GH3-1 and GH8-5), were grown at Clayton, NC, in both 2017 and 2018. The experimental design was identical in both years, as the plants were arranged in 9-plant rows, with each row containing one individual of each genotype in random order. While 35 replications of the 9-plant rows were grown in 2017, 70 replications were planted in 2018. Each plant represented an experimental unit. In these experiments, flowering time was defined as the date at which floral buds first emerged. Once the first floral structures began to appear, the field was monitored three times a week. Plants with floral buds were topped and the date recorded. Topped plants were subsequently treated with the contact-localized suckercide FluPro^®^ in accordance with the manufacturer’s instructions (Chemtura Agro Solutions, USA). Total aerial biomass was measured at harvest, and the plants were air-cured in a barn for 10 weeks. After curing, the fourth leaf from the top was removed, and its lamina was stripped from the mid-rib. The leaf was then dried to completion in a paper bag and ground to a fine powder for chemical analysis.

### Nitrate, alkaloid, and TSNA analysis

Ground leaf tissue was analyzed for nitrate content by using a Lachet QuikChem 8500 instrument in accordance to the manufacturer’s protocol (Lachat QuikChem method 12-107-04-1-J, Lachet Instruments, Loveland, CO, USA). Alkaloid and TSNA assays were conducted at the University of Kentucky tobacco analytical lab. Quantification of the alkaloids nicotine, nornicotine, anatabine, and anabasine was accomplished by using a Perkin–Elmer Autosystem XL Gas Chromatograph as described previously^10^. Total alkaloid levels were calculated as the sum of the levels of the four individual alkaloids. NNN, NNK, NAT, and NAB were extracted and quantified by following “Method 1” of Morgan *et al*^42^. Total TSNA levels were defined as the sum of the NNN, NNK, NAT, and NAB levels.

### Statistical analysis

The GLM procedure of SAS 9.4 (SAS Institute, Cary, NC) was used to perform analysis of variance (ANOVA) and compare the mean values of the test lines in this study. Multiple comparisons of means were conducted in accordance with the Ryan–Einot–Gabriel–Welsch (REGWQ) multiple range test. Appropriate power-transformations were performed for measurements of some traits to equalize variances and/or normalize distributions. Means and standard errors in their original scales are displayed in tables and figures; but, the labels of groupings are the results from analysis of transformed data. A log-rank test was used to compare the flowering time of the transgenic and WT lines included in this study. Days from transplanting to flowering were used in time-to-event analysis. Plants that had not flowered by harvest were treated as having censored time to flowering. Owing to the large numbers of censored data in certain genotypes, comparisons were summarized with groupings under Bonferroni correction. Overall and pairwise comparisons of each line’s time to flowering was performed by using the LIFETEST procedure of SAS 9.4 (SAS Institute, Cary, NC). The FREQ procedure with the CHISQ option (SAS9.4) was used in χ^2^ test to analyze the segregation of transgenes in T_1_ progeny.

## Supplementary Information

Supplementary Information.
