# Supplementary material for: Constitutive activation of nitrate reductase in tobacco alters flowering time and plant biomass
Source: Sci Rep. 2021 Feb 19;11:4222. doi: 10.1038/s41598-021-83797-7 (PMC7896089; doi:10.1038/s41598-021-83797-7)
Supplement: Supplementary file 1 — Supplementary Information. [file 41598_2021_83797_MOESM1_ESM.docx]

**SUPPLEMENTARY INFORMATION**

**Constitutive Activation of Nitrate Reductase in Tobacco Alters Flowering Time and Plant Biomass**

**Jianli Lu^a^, Niharika N. Chandrakanth^a^, Ramsey S. Lewis^a^, Karen Andres^a^, Lucien Bovet^b^, Simon Goepfert^b^, and Ralph E. Dewey^a,*^**

| **Supplementary Table S1. Effects of fertilization on tobacco height, biomass, flowering time and number of harvestable leaves across different genetic backgrounds.** | | | | | | | | | | |
| --- | --- | --- | --- | --- | --- | --- | --- | --- | --- | --- |
| **Genotype** | **Fertilization** | **Young Plants** (07/27/15) | | |  | **Mature Plants** (10/07/15) | | | | |
|  |  | **N** | **Height (cm)^*^** | **Above-soil biomass (g)** |  | **N** | **Average flowering date** | **Height (cm)^**^** | **Above-soil biomass (g)** | **Harvestable leaf #** |
| 35S:S523D-NR | 19 mM NO_3_ | 12 | 10.0±1.3^A^ | 177±18.1^A^ |  | 12 | Aug 23 ± 2.4 days^A^ | 83.3±8.9^A^ | 762±26.9^A^ | 19.8±1.5^A^ |
|  | 8 mM NO_3_ | 12 | 8.4±0.7^B^ | 119±8.2^B^ |  | 10 | Aug 31 ± 2.4 days^B^ | 83.0±6.0^A^ | 594±33.7^B^ | 21.4±1.2^B^ |
| TN90e4e5 | 19 mM NO_3_ | 12 | 9.7±0.9^a^ | 182±19.1^a^ |  | 12 | Aug 30 ± 3.5 days^a^ | 93.7±9.9^a^ | 866±64.2^a^ | 23.8±2.7^a^ |
|  | 8 mM NO_3_ | 12 | 8.1±1.0^b^ | 124±8.9^b^ |  | 12 | Sep 9 ± 4.8 days^b^ | 88.6±12.3^a^ | 637±60.5^b^ | 25.2±2.3^a^ |
| Means ± standard deviations with the same letter are not significantly different at α=0.05 according to REGWQ grouping.  *Height measured at apical meristem  **Height measured at site of topping | | | | | | | | | | |


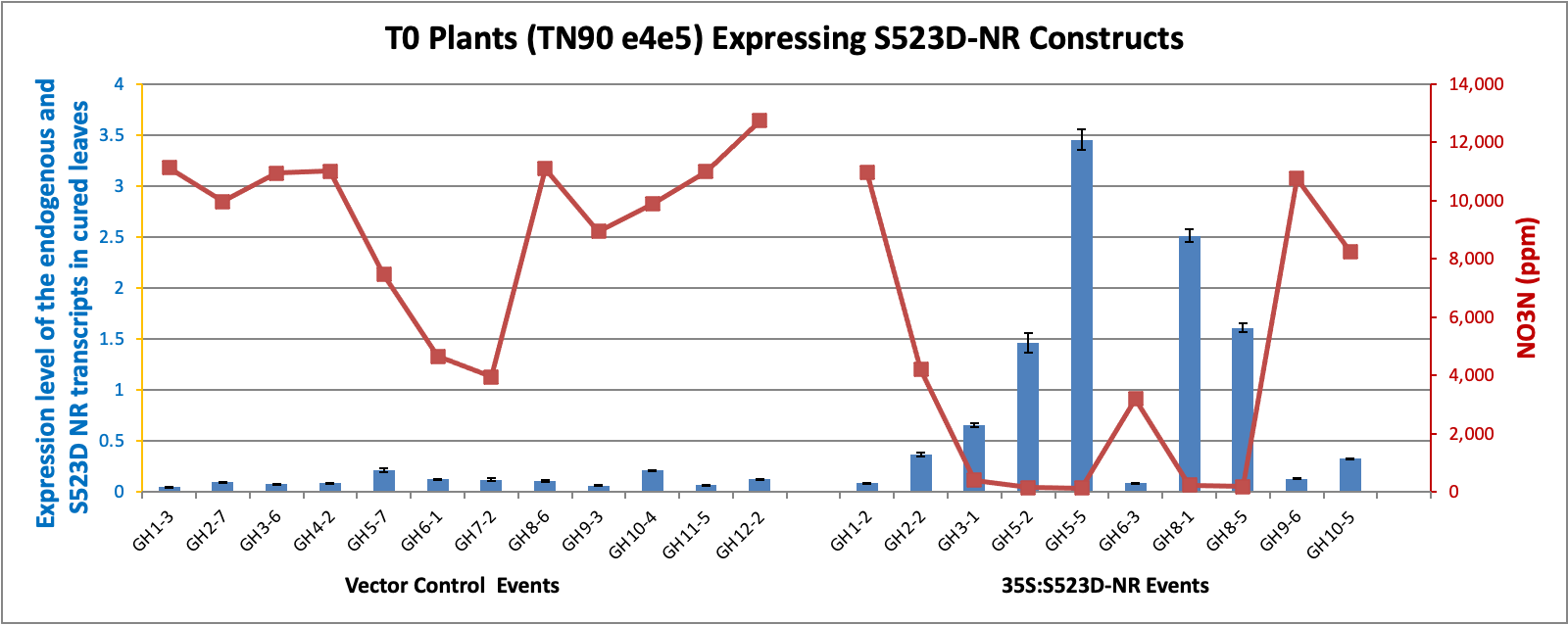


**Supplementary Fig. S1.** Quantitative RT-PCR (qRT-PCR) and nitrate analysis of T_0_ TN90 e4e5 plants transformed with vector control or 35S:S523D-NR constructs. qRT-PCR results are displayed with blue bars using the values on the left. Data represent the mean ± standard error of three replications. Nitrate results are shown as red boxes using the scale on the right.


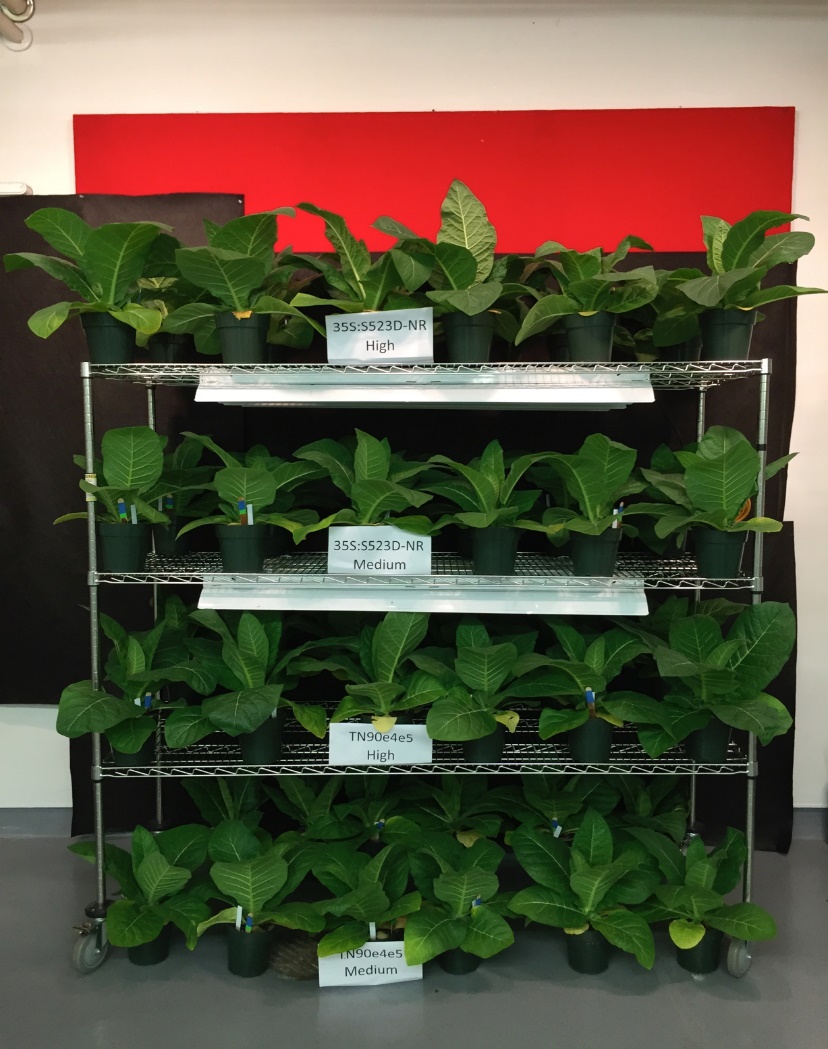


**Supplementary Fig. S2** Phytotron-grown plants included in the first harvest.

Plants were provided either a medium (8 mM) or high (19 mM) level of N

fertilization

**
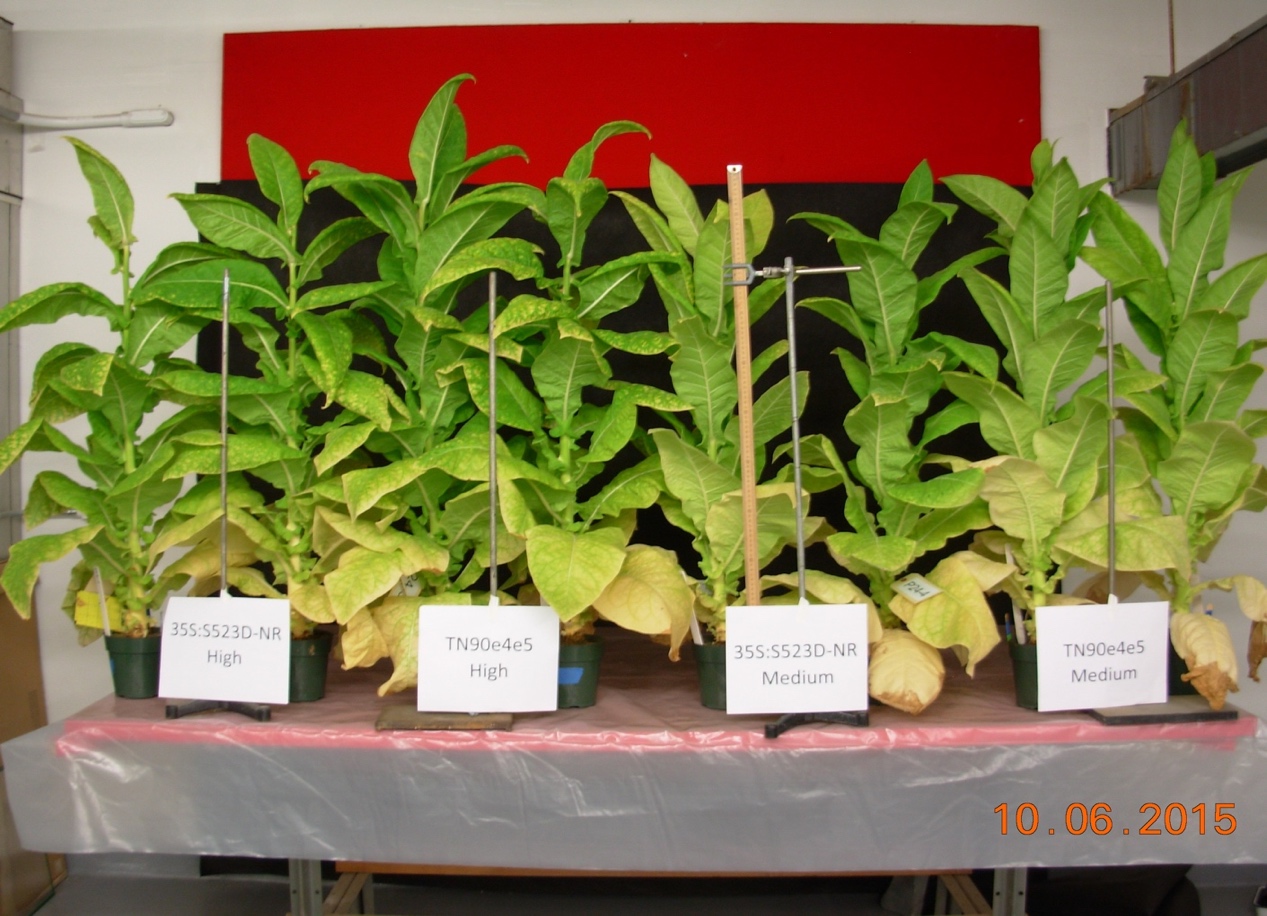
**

**Supplementary Fig. S3** Representative subsample of the Phytotron-grown plants included in the final harvest. Plants were provided either a medium (8 mM) or high (19 mM) level of N

fertilization
